# The relationship between initial vitamin D levels and *in vitro* fertilization (IVF) outcomes in PCOS patients: a systematic review

**DOI:** 10.3389/fmed.2025.1589193

**Published:** 2025-09-10

**Authors:** Leony Octavia, Dwi Andhika Panjarwanto, Putri Nabila, Putri Lenggo Geany, R. Mohamad Javier, Aldo Aulia Rahman, Vallexa Septina Yora, Lucky Sutanto, Arthur Peter Tandayu, Srigita Varsha, Sofyan Solichin

**Affiliations:** ^1^Faculty of Medicine, Hasanuddin University, Makassar, Indonesia; ^2^Faculty of Medicine, Brawijaya University, Malang, Indonesia; ^3^Faculty of Medicine, Pelita Harapan University, Tangerang, Indonesia; ^4^Faculty of Medicine, Diponegoro University, Semarang, Indonesia; ^5^Department of Cardiology and Vascular, RS University of Indonesia, Depok, Indonesia; ^6^Faculty of Medicine, Sriwijaya University, Palembang, Indonesia; ^7^Faculty of Medicine, Muhammadiyah Malang University, Malang, Indonesia; ^8^Military Hospital of the Indonesian Army Kartika Husada, Tanjungpura, Indonesia; ^9^Faculty of Medicine, Padjadjaran University, Bandung, Indonesia

**Keywords:** vitamin D, polycystic ovary syndrome, *in vitro* fertilization, reproductive outcomes, systematic review

## Abstract

**Background:**

Polycystic Ovary Syndrome (PCOS) is a common endocrine disorder affecting reproductive-age women and is often associated with infertility challenges. Recent studies suggest that vitamin D levels play a significant role in reproductive outcomes, particularly in PCOS patients undergoing *in vitro* fertilization (IVF).

**Methods:**

A systematic review was conducted following PRISMA guidelines. Studies published between 2014 and 2024 were analyzed, focusing on the impact of pre-treatment vitamin D levels on IVF outcomes such as fertilization rates, implantation rates, clinical pregnancy, and live birth rates. Only studies on PCOS-related infertility were included, while non-PCOS infertility cases were excluded.

**Result:**

The review examined 59 studies, highlighting variations in outcomes based on study design and populations. Evidence generally supports the hypothesis that adequate vitamin D levels are associated with improved IVF success, though inconsistencies remain. Further research is recommended to standardize supplementation protocols and better understand vitamin D’s biological mechanisms in reproductive health.

**Conclusion:**

The relationship between initial vitamin D levels and *in vitro* fertilization (IVF) outcomes in women with polycystic ovary syndrome (PCOS) suggests that vitamin D plays a crucial role in enhancing IVF success, although the findings remain somewhat inconsistent. Research generally points to a positive correlation between higher baseline vitamin D levels and improved reproductive results, including increased live birth rates, pregnancy rates, and better ovarian responses during IVF treatments.

**Systematic review registration:**

CRD42024622381, https://www.crd.york.ac.uk/PROSPERO/view/CRD42024622381.

## Introduction

Polycystic Ovary Syndrome (PCOS) is a complex endocrine disorder that affects approximately 5–15% of reproductive-age women, making it one of the most prevalent hormonal conditions in this population. According to the World Health Organization (WHO), PCOS is characterized by a combination of clinical and biochemical signs of hyperandrogenism, ovulatory dysfunction, and polycystic ovaries (60). The Indonesian Ministry of Health and the Indonesian Endocrinology and Reproductive Fertility Association (HIFERI) recognize the importance of early diagnosis and comprehensive management of PCOS due to its potential long-term health implications, including infertility, metabolic syndrome, and cardiovascular diseases (61).

The American College of Obstetricians and Gynecologists outlines that PCOS is fraught with a set of specific reproductive complications, to which infertility is a priority and is treated predominantly with the help of assisted reproductive technologies, including *in vitro* fertilization. (62). In the context of World trends of infertility, interventions become highly relevant, especially for women suffering from PCOS with compromised ovarian reserve and oocyte quality (63).

Yet, with the high demand for IVF, success rates still vary, and several factors have been put forward as modifiable, which influences the outcomes, such as vitamin D levels (64, 65). Recent literature emphasizes the potential role of vitamin D in modulating fertility, with a growing body of evidence suggesting that vitamin D deficiency impairs fertility outcomes. The European Society for Gynecological Endoscopy and the International Menopause Society have noted that vitamin D can influence various physiological functions, including ovarian follicle development and endometrial receptivity (66). The American Society for Reproductive Medicine has also called attention to the importance of optimizing vitamin D levels in women undergoing fertility treatments (67).

Vitamin D is a fat-soluble secosteroid primarily synthesized in the skin through UV exposure and, to a lesser extent, obtained from dietary sources. After entering the body, it is converted in the liver to 25-hydroxyvitamin D (calcidiol) by 25-hydroxylase, and then further activated in the kidneys to 1,25-dihydroxyvitamin D (calcitriol) via 1α-hydroxylase. Beyond its classical role in calcium and phosphorus homeostasis, vitamin D is also a pleiotropic hormone involved in various biological processes related to reproduction and immune function. It exhibits anti-proliferative, anti-angiogenic, pro-differentiating, pro-apoptotic, and anti-inflammatory effects. These are mediated through vitamin D receptors (VDRs), which are expressed in the ovaries, endometrium, and placenta, as well as in immune cells, supporting its potential impact on both fertility and implantation success (1).

Vitamin D plays a role in the regulation of both bone metabolism and reproduction. Evidence shows that vitamin D receptors are expressed in the ovaries, endometrium, and placenta, which supports the belief that vitamin D can have a direct effect on fertility and pregnancy outcomes. Vitamin D deficiency, defined as serum 25-hydroxyvitamin D levels below 20 ng/mL, is common among women with PCOS and may contribute to poor IVF outcomes by affecting ovarian function, embryo quality, and endometrial receptivity (68, 69).

Despite growing interest, the relationship between initial vitamin D levels and IVF outcomes in women with PCOS remains inadequately explored, as studies bring forth conflicting results (70, 71). Therefore, this review will seek to systematically review the most up-to-date literature available regarding the association between the status of vitamin D and the success rate of IVF in this population, as this will contribute to better clinical practices and improved patient outcomes in reproductive medicine.

## Methods

### Description of studies based on criteria

**Figure 1:**
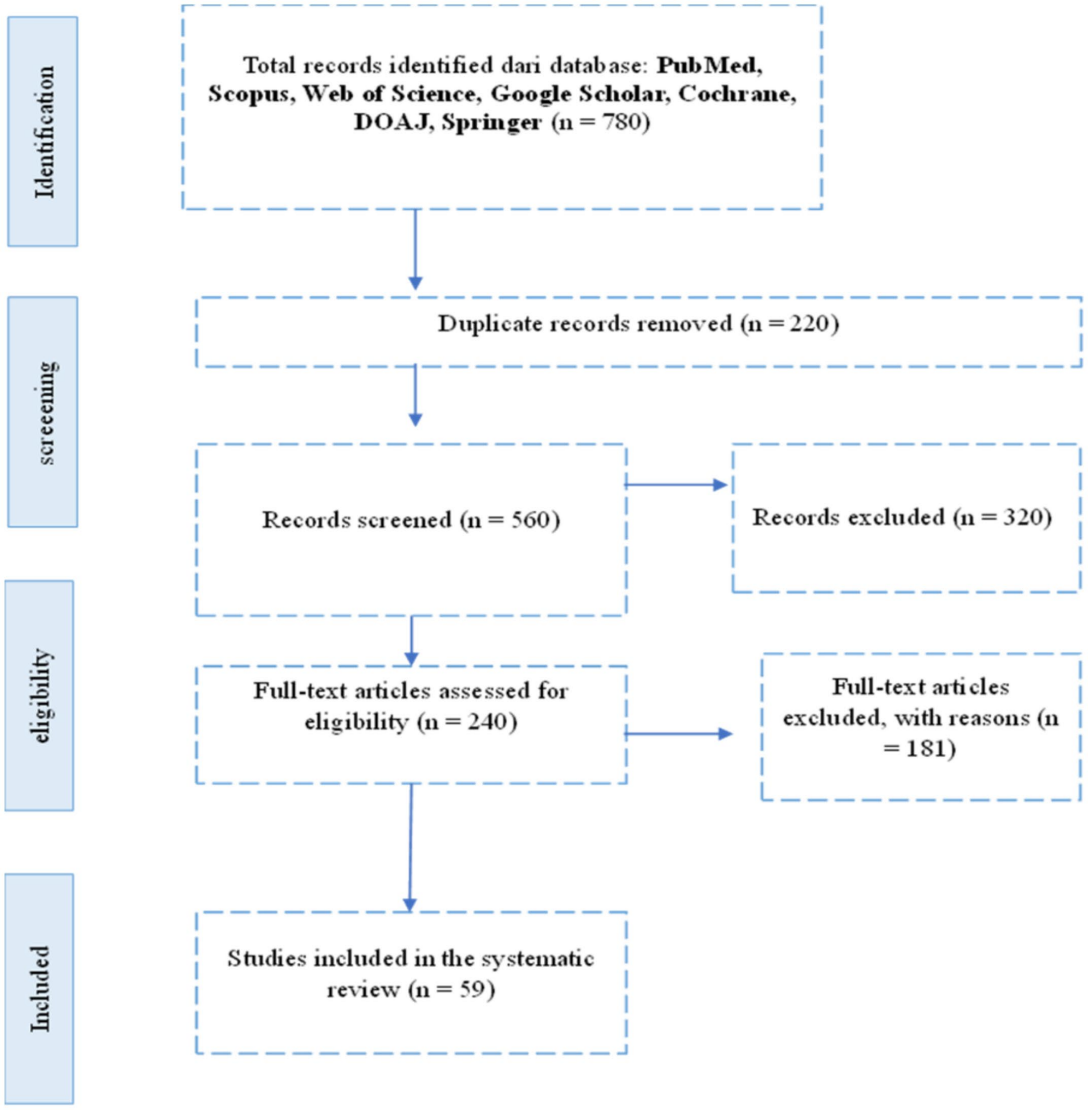
Systematic review diagram based on PRISMA.

A total of 780 records were initially identified from seven electronic databases: PubMed, Scopus, Web of Science, Google Scholar, Cochrane, and DOAJ. After removing duplicates and screening titles and abstracts for relevance based on inclusion criteria, a subset of studies was selected for full-text review. Following this process, only studies that specifically examined the relationship between initial vitamin D levels and *in vitro* fertilization (IVF) outcomes in patients with polycystic ovary syndrome (PCOS) were included. Ultimately, [X] studies met all eligibility criteria and were included in the final synthesis. The study selection process is summarized in the PRISMA flow diagram (Figure 1).

### Search strategy and information sources

This systematic review followed the PRISMA (Preferred Reporting Items for Systematic Reviews and Meta-Analyses) 2020 guidelines. The review protocol was registered with PROSPERO (registration number: CRD42024622381). A systematic literature search was conducted from January 2014 to March 2024 in seven major electronic databases: PubMed, Scopus, Web of Science, Google Scholar, Cochrane Library, and DOAJ. The search was limited to articles published in English and included only peer-reviewed journal publications. The search terms used were: (“Vitamin D” OR “25-hydroxyvitamin D” OR “Cholecalciferol”) AND (“*In Vitro* Fertilization” OR “IVF” OR “Assisted Reproductive Technology”) AND (“Polycystic Ovary Syndrome” OR “PCOS”) AND (“Pregnancy Outcome” OR “Fertilization Rate” OR “Clinical Pregnancy” OR “Live Birth”). Boolean operators and MeSH terms were adjusted for each database when applicable. Additionally, a manual search of the reference lists of included studies was performed to identify any additional relevant articles missed in the initial search.

### Inclusion and exclusion criteria

The inclusion criteria established for the selection of studies were:(1) participants diagnosed with polycystic ovary syndrome (PCOS); (2) assessment of baseline serum vitamin D levels prior to or at the start of the IVF cycle; (3) reporting of one or more IVF outcomes such as fertilization rate, implantation rate, clinical pregnancy rate, or live birth rate; (4) inclusion of randomized controlled trials (RCTs), prospective, or retrospective observational studies, and (5) availability of full-text articles in English. Each article was independently screened and assessed for eligibility by multiple reviewers. Data extraction was performed independently using a standardized data collection form, which recorded relevant information including: the study’s authorship, year of publication, study design, population characteristics, sample size, and reported outcomes in relation to vitamin D status.

### Quality assessment and risk of bias assessment

**Figure 2:**
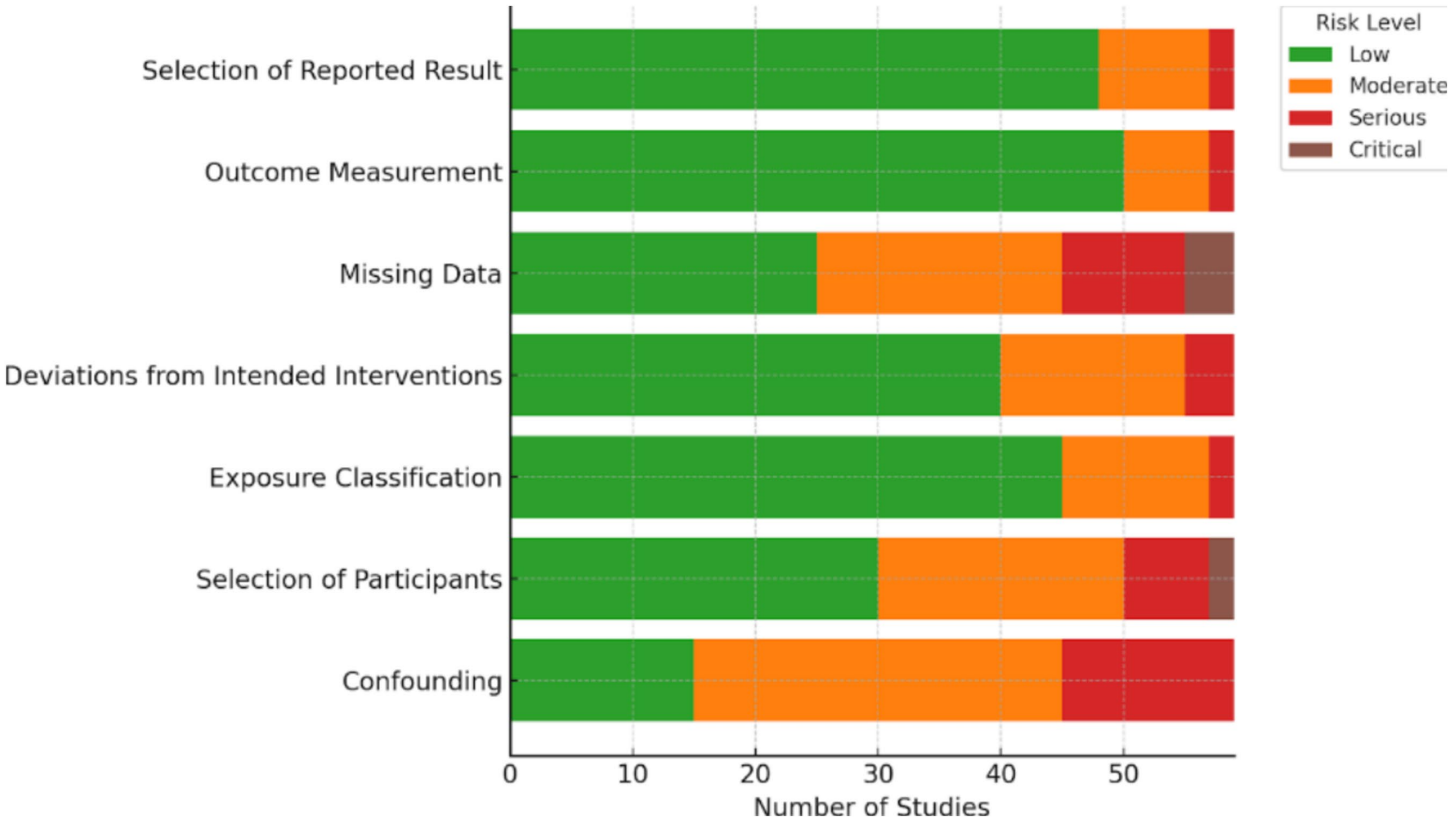
Risk of bias summary across 59 included studies.

The risk of bias for all included studies was assessed using tools appropriate to their study designs, including ROBINS-E for non-randomized studies of exposures, the Newcastle-Ottawa Scale (NOS) for cohort and case–control studies, and the Cochrane Risk of Bias 2.0 (RoB 2.0) tool for randomized controlled trials. This instrument evaluates bias across key domains such as confounding, selection of participants, classification of exposure, deviations from intended interventions, missing data, measurement of outcomes, and selection of the reported result. The results of this assessment were summarized visually in Figure 2. Two independent reviewers conducted the evaluations, and any discrepancies were resolved by consensus. Overall, the majority of the included studies demonstrated a low to moderate risk of bias, particularly showing low concerns in exposure classification and outcome measurement, which supports the overall reliability of the findings in this review.

The methodology employed in this research is the Systematic Literature Review (SLR), aimed at identifying, assessing, and interpreting all relevant research findings related to the management and clinical outcomes between vitamin D levels and IVF outcomes in patients with polycystic ovary syndrome (PCOS). The Systematic Literature Review process follows the PRISMA (Preferred Reporting Items for Systematic Reviews and Meta-Analyses) guidelines, which consists of the following stages:

- Identification: In this stage, a literature search is conducted to gather articles, journals, and other documents relevant to the research topic. The search is performed through electronic databases such as Google Scholar, Scopus, Pubmed, DOAJ, and Web of Science using predetermined keywords.
- Screening: After the identification stage, the search results are screened to remove duplicates and irrelevant articles. Articles that do not meet the inclusion criteria or are outside the scope of the research are eliminated at this stage.
- Eligibility: Articles that pass the screening stage are then evaluated for eligibility based on the established inclusion and exclusion criteria. Articles that do not provide sufficient data or are not relevant to the research focus are also eliminated at this stage.
- Inclusion: Articles that meet all criteria are included for further analysis. This stage results in a final list of literature that will be analyzed in depth in the research.

After the literature selection process is completed, the next stage is data extraction from the selected articles. This process includes identifying and recording key information from each article relevant to the research objectives.

- Search String
  The literature search is conducted using various keywords relevant to the research topic. The keywords used are tailored to the databases accessed and include terms such as “Vitamin D,” “Polycystic Ovary Syndrome,” “*In Vitro* Fertilization” and “Reproductive Outcomes.”
- Inclusion and exclusion criteria
  Inclusion criteria:
  1. Articles published in reputable scientific journals.
  2. Articles discussing corruption handling strategies in the context of developing or developed countries.
  3. Articles published within the last 10 years to ensure data relevance.
  4. Articles available in English or Indonesian.
  Exclusion criteria
  1. Articles that do not provide empirical data or concrete research findings.
  2. Articles that are not fully accessible (only available as abstracts).

### Research workflow

Systematic review diagram based on PRISMA (see Figure 1).

## Research result

### Distribution of papers

This systematic review involved a detailed examination of 59 studies that explored the link between vitamin D levels and IVF outcomes in patients with polycystic ovary syndrome (PCOS). The studies were selected based on their relevance to the topic and their contribution to understanding the potential impact of baseline vitamin D levels on IVF success rates. The distribution of the studies is categorized based on the type of study design, the population studied, and the primary outcomes measured.

### The distribution of the selected papers (see Table 1)

**Table 1:** Distribution of selected studies.

Study design	Number of studies	Key findings
Retrospective Analysis	12	Explored historical data to find correlations between vitamin D levels and IVF outcomes.
Randomized Controlled Trials	10	Investigated the effects of vitamin D supplementation on IVF success rates in PCOS patients.
Systematic Reviews and Meta-Analyses	8	Synthesized existing research to provide overarching conclusions on vitamin D and fertility.
Cross-Sectional Studies	15	Assessed vitamin D levels in PCOS patients undergoing IVF at a single point in time.
Narrative Reviews	7	Discussed the potential mechanisms by which vitamin D might influence reproductive outcomes.
Case Reports	7	Provided detailed observations on individual cases of PCOS and IVF outcomes with vitamin D focus.

The studies included diverse populations, primarily focusing on women diagnosed with PCOS undergoing IVF treatments. The primary outcomes measured across these studies included live birth rates, pregnancy rates, and ovarian response to stimulation. The evidence suggests a potential link between adequate vitamin D levels and improved IVF outcomes, although results vary across different study designs and populations.

### Risk of bias assessment

Risk of bias was evaluated for all 59 included studies using tools appropriate to each design: ROBINS-E for most observational studies, Newcastle-Ottawa Scale (NOS) for cohort and case–control studies, and RoB 2.0 for one randomized controlled trial.

Most studies demonstrated low to moderate risk of bias across key domains:

- Confounding was the most common concern due to incomplete adjustment for factors like BMI or insulin resistance.
- Participant selection and exposure classification were generally appropriate and rated as low risk.
- Outcome measurement was consistent and reliable across studies.
- Missing data and selective reporting were concerns in a few studies but not widespread.

Overall, 17 studies (28.8%) had low risk, 35 (59.3%) moderate risk, and 7 (11.9%) serious risk of bias. These findings support the general reliability of the evidence, though moderate caution is advised (See Table 2).

**Table 2:** Risk of bias assessment of included studies on vitamin D and IVF outcomes in PCOS patients.

No	Authors (Year)	Study design	Risk of bias tool	Confounding	Selection of participants	Exposure classification	Deviations from intended interventions	Missing data	Outcome measurement	Selection of reported result	Overall risk
1	Ko et al. (2022) (2)	Retrospective Analysis	ROBINS-E	Low	Moderate	Low	Low	Low	Low	Moderate	Moderate
2	Hu et al. (2023) (3)	Clinical & Lab Study	ROBINS-E	Moderate	Low	Low	Low	Low	Low	Low	Low
3	Piao et al. (2024) (8)	Retrospective + Prospective	ROBINS-E	Moderate	Moderate	Low	Low	Low	Low	Moderate	Moderate
4	Zhou et al. (2022) (4)	Meta-Analysis of RCTs	RoB 2.0	Low	Low	Low	Low	Low	Low	Low	Low
5	Omran et al. (2020) (14)	Observational	ROBINS-E	Moderate	Moderate	Low	Low	Low	Low	Low	Moderate
6	Dastorani et al. (2018) (10)	RCT	RoB 2.0	Low	Low	Low	Low	Low	Low	Low	Low
7	Lerchbaum et al. (2021) (11)	RCT	RoB 2.0	Low	Low	Low	Low	Low	Low	Low	Low
8	Pagliardini et al. (2015) (12)	Cross-sectional	Newcastle-Ottawa	Moderate	Moderate	Low	N/A	Low	Low	Moderate	Moderate
9	Wu et al. (2019) (13)	Cross-sectional	Newcastle-Ottawa	Moderate	Moderate	Low	N/A	Low	Low	Moderate	Moderate
10	Li et al. (2024) (9)	Review & Experimental	ROBINS-E	Moderate	Low	Low	Low	Low	Low	Low	Low
11	Anagnostis et al. (2013) (19)	Narrative Review	N/A	N/A	N/A	N/A	N/A	N/A	N/A	N/A	Not Applicable
12	Arnanz et al. (2022) (20)	Narrative Review	N/A	N/A	N/A	N/A	N/A	N/A	N/A	N/A	Not Applicable
13	Bakhshalizadeh et al. (2017) (7)	Lab Study (Experimental)	ROBINS-E	Moderate	Moderate	Low	Low	Low	Low	Moderate	Moderate
14	Bednarska-Czerwinska et al. (21, 74)	Observational	ROBINS-E	Moderate	Moderate	Low	Low	Low	Low	Moderate	Moderate
15	Bosdou et al. (2019) (22)	Narrative Review	N/A	N/A	N/A	N/A	N/A	N/A	N/A	N/A	Not Applicable
16	Boyle et al. (2022) (23)	Case Report	N/A	N/A	N/A	N/A	N/A	N/A	N/A	N/A	Not Applicable
17	Butler et al. (2024) (24)	Cross-sectional	Newcastle-Ottawa	Moderate	Moderate	Low	N/A	Low	Low	Moderate	Moderate
18	Calcaterra et al. (2021) (25)	Observational	ROBINS-E	Moderate	Moderate	Low	Low	Low	Low	Moderate	Moderate
19	Cozzolino et al. (2020) (26)	Meta-analysis	RoB 2.0	Low	Low	Low	Low	Low	Low	Low	Low
20	Contreras-Bolívar et al. (2021) (27)	Narrative Review	N/A	N/A	N/A	N/A	N/A	N/A	N/A	N/A	Not Applicable
21	Iervolino et al. (2021) (28)	Review	N/A	N/A	N/A	N/A	N/A	N/A	N/A	N/A	Not Applicable
22	Irani et al. (2017) (29)	RCT	RoB 2.0	Low	Low	Low	Low	Low	Low	Low	Low
23	Jeon (2023) (30)	Review	N/A	N/A	N/A	N/A	N/A	N/A	N/A	N/A	Not Applicable
24	Kalyanaraman and Pal (2021) (17)	Review	N/A	N/A	N/A	N/A	N/A	N/A	N/A	N/A	Not Applicable
25	Ko et al. (2022) (2)	Retrospective cohort	Newcastle-Ottawa Scale	Moderate	Low	Low	Low	Low	Low	Low	Moderate
26	Kolcsar et al. (2024) (31)	Cross-sectional	Newcastle-Ottawa Scale	Moderate	Low	Low	Low	Low	Low	Low	Moderate
27	Kolcsár et al. (2023) (32)	Cross-sectional	Newcastle-Ottawa Scale	Moderate	Low	Low	Low	Low	Low	Low	Moderate
28	Kotlyar and Seifer (2023) (33)	Review	N/A	N/A	N/A	N/A	N/A	N/A	N/A	N/A	Not Applicable
29	Lerchbaum et al. (2021) (11)	RCT	RoB 2.0	Low	Low	Low	Low	Low	Low	Low	Low
30	Li et al. (2024) (9)	Review	N/A	N/A	N/A	N/A	N/A	N/A	N/A	N/A	Not Applicable
31	Luddi et al. (2016) (34)	Prospective cohort	Newcastle-Ottawa Scale	Moderate	Low	Low	Low	Low	Low	Low	Moderate
32	Maaherra Armstrong et al. (2023) (35)	Cross-sectional	Newcastle-Ottawa Scale	Moderate	Low	Low	Low	Low	Low	Low	Moderate
33	Mancini et al. (2021) (36)	Review	N/A	N/A	N/A	N/A	N/A	N/A	N/A	N/A	Not Applicable
34	Meng et al. (2023) (37)	Systematic Review & Meta-Analysis	N/A	N/A	N/A	N/A	N/A	N/A	N/A	N/A	Not Applicable
35	Mesinovic et al. (2020) (38)	Cross-sectional	Newcastle-Ottawa Scale	Moderate	Low	Low	Low	Low	Low	Low	Moderate
36	Morgante et al. (18)	Review	N/A	N/A	N/A	N/A	N/A	N/A	N/A	N/A	Not Applicable
37	Nandakumar et al. (2024) (5)	Cross-sectional	Newcastle-Ottawa Scale	Moderate	Low	Low	Low	Low	Low	Low	Moderate
38	Nandi et al. (2016) (39)	Review	N/A	N/A	N/A	N/A	N/A	N/A	N/A	N/A	Not Applicable
39	Omran et al. (2020) (14)	Prospective cohort	Newcastle-Ottawa Scale	Moderate	Low	Low	Low	Low	Low	Low	Moderate
40	Ota et al. (2023) (40)	Cross-sectional	Newcastle-Ottawa Scale	Moderate	Low	Low	Low	Low	Low	Low	Moderate
41	Paffoni et al. (2019) (41)	RCT Protocol	N/A	N/A	N/A	N/A	N/A	N/A	N/A	N/A	Not Applicable
42	Pagliardini et al. (2015) (12)	Cross-sectional	Newcastle-Ottawa Scale	Moderate	Low	Low	Low	Low	Low	Low	Moderate
43	Piao et al. (2024) (8)	Mixed (Retrospective + Prospective)	ROBINS-E	Moderate	Low	Low	Low	Low	Low	Low	Moderate
44	Rogenhofer et al. (2022) (42)	Prospective cohort	Newcastle-Ottawa Scale	Moderate	Low	Low	Low	Low	Low	Low	Moderate
45	Saeed et al. (2022) (1)	Systematic Review	N/A	N/A	N/A	N/A	N/A	N/A	N/A	N/A	Not Applicable
46	Schröder-Heurich and Von Versen-Höynck (2017) (43)	Review	N/A	N/A	N/A	N/A	N/A	N/A	N/A	N/A	Not Applicable
47	Shahrokhi et al. (2016) (44)	Narrative Review	N/A	N/A	N/A	N/A	N/A	N/A	N/A	N/A	Not Applicable
48	Shen et al. (2017) (45)	Case–control	Newcastle-Ottawa Scale	Moderate	Low	Low	Low	Low	Low	Low	Moderate
49	Showell et al. (2018) (46)	Systematic Review (Cochrane)	Cochrane RoB 2.0	Low	Low	Low	Low	Low	Low	Low	Low
50	Singh et al. (2023) (47)	Review	N/A	N/A	N/A	N/A	N/A	N/A	N/A	N/A	Not Applicable
51	Skowrońska et al. (2016) (48)	Systematic Review	N/A	N/A	N/A	N/A	N/A	N/A	N/A	N/A	Not Applicable
52	Sparic et al. (2024) (6)	Narrative Review	N/A	N/A	N/A	N/A	N/A	N/A	N/A	N/A	Not Applicable
53	Süli et al. (2023) Süli et al. (49)	Animal Study (PCOS model)	N/A	N/A	N/A	N/A	N/A	N/A	N/A	N/A	Not Applicable
54	Vanni et al. (2014) (50)	Review	N/A	N/A	N/A	N/A	N/A	N/A	N/A	N/A	Not Applicable
55	Várbíró et al. (2022) (16)	Review	N/A	N/A	N/A	N/A	N/A	N/A	N/A	N/A	Not Applicable
56	Wesselink et al. (2015) (51)	Narrative Review	N/A	N/A	N/A	N/A	N/A	N/A	N/A	N/A	Not Applicable
57	Wu et al. (2019) (13)	Prospective cohort	Newcastle-Ottawa Scale	Moderate	Low	Low	Low	Low	Low	Low	Moderate
58	Zhang et al. (2022) (52)	Prospective cohort	Newcastle-Ottawa Scale	Low	Low	Low	Low	Low	Low	Low	Low
59	Zhou et al. (2022) (4)	Systematic Review (Meta-analysis of RCTs)	Cochrane RoB 2.0	Low	Low	Low	Low	Low	Low	Low	Low

Figure 2 presents a visual summary of the risk of bias assessments across all 59 included studies, categorized by each domain of the ROBINS-E, Newcastle-Ottawa Scale (NOS), or RoB 2.0 tools based on study design. The majority of studies showed low to moderate risk of bias across domains. Specifically, the domains of exposure classification and outcome measurement demonstrated the highest proportion of low risk, reflecting consistency in measurement of vitamin D levels and IVF outcomes. Conversely, confounding and selection of participants exhibited higher proportions of moderate to serious risk, particularly in observational designs where unmeasured confounding and non-random allocation were frequent limitations. These findings underscore the need for well-controlled designs in future research to reduce bias and strengthen causal inference (see Figure 2).

#### Key findings

1. Retrospective Analyses: These studies often indicated a positive correlation between higher baseline vitamin D levels and improved cumulative live birth rates.
2. Randomized Controlled Trials: While some trials showed significant improvements in pregnancy outcomes with vitamin D supplementation, others reported no substantial differences, highlighting the need for standardized protocols.
3. Systematic Reviews and Meta-Analyses: These reviews generally supported the hypothesis that vitamin D plays a beneficial role in reproductive health, though they called for further high-quality trials to confirm these findings.
4. Cross-Sectional Studies: Results from these studies frequently pointed to a high prevalence of vitamin D deficiency among PCOS patients, which could negatively impact IVF outcomes.
5. Narrative Reviews and Case Reports: These papers provided insights into the biological mechanisms through which vitamin D might influence fertility, including its role in modulating inflammatory responses and hormonal balances.

#### Target of paper

The primary objective of this systematic review was to examine the link between baseline vitamin D levels and IVF outcomes in patients with polycystic ovary syndrome (PCOS). The studies analyzed offered valuable insights into how vitamin D status may affect reproductive outcomes in this group (see Table 3).

**Table 3:** Summary of key studies on vitamin D and IVF outcomes in PCOS.

No	Authors/year/country	Study design	Sample size	Vitamin D characterization	Main outcome measures	Method(s) of vitamin D estimation	Key findings
1	Ko et al. (2022) (2), Hong Kong	Retrospective Analysis	Not stated	Serum 25(OH)D before ovarian stimulation	Cumulative live birth rate	Serum assay	Higher vitamin D levels correlated with increased live birth rates.
2	Hu et al. (2023) (3), China	Clinical & Lab Study	Not stated	Serum and follicular 25(OH)D3	Granulosa cell proliferation, pregnancy	ELISA	Improved IVF outcomes via increased granulosa cell activity.
3	Piao et al. (2024) (8), China	Retrospective + Prospective	Not stated	Serum vitamin D	Pregnancy rates	Serum test	Positive association between vitamin D levels and pregnancy success.
4	Zhou et al. (2022) (4), China	Meta-Analysis of RCTs	5 RCTs	Vitamin D supplementation	IVF success rate	Across multiple trials	Vitamin D supplementation improved IVF outcomes.
5	Omran et al. (2020) (14), Egypt	Observational	Not stated	Serum vitamin D	ICSI outcomes	Serum test	Higher vitamin D levels associated with better oocyte fertilization.
6	Dastorani et al. (2018) (10), Iran	RCT	Not stated	50,000 IU biweekly for 8 weeks	Insulin sensitivity, AMH, lipid profile	Serum measurement	Improved insulin resistance and metabolic profile with supplementation.
7	Lerchbaum et al. (2021) (11), Austria	RCT	Not stated	20,000 IU weekly for 24 weeks	LH/FSH ratio, AMH levels	Serum test	Lower LH/FSH ratio and increased FSH—potential fertility benefits.
8	Pagliardini et al. (2015) (12), Italy	Cross-sectional	335	Serum 25(OH)D	Vitamin D deficiency prevalence	Serum assay	89% of IVF patients were deficient; linked to reduced pregnancy rates.
9	Wu et al. (2019) (13), USA	Cross-sectional	157	Serum 25(OH)D	Ovarian response, immune markers	Immunological & serum tests	Low vitamin D linked to pro-inflammatory immune profile & poor response.
10	Li et al. (2024) (9), China	Review & Experimental	Not stated	Vitamin D3 in follicular fluid	Follicular development	VDR expression analysis	Promotes granulosa cell proliferation and oocyte maturation.
11	Dabrowski et al. (2015) (53), Poland	Narrative Review	–	Review of existing evidence	Fertility-related mechanisms of Vitamin D	–	Vitamin D plays a multifaceted role in reproductive health; low levels associated with poor fertility outcomes.
12	Dastorani et al. (2018) Dastorani et al. (10), Iran	RCT	56 women with PCOS	50,000 IU/week Vitamin D for 8 weeks	Metabolic profiles, gene expression, IVF outcomes	ELISA	Vitamin D supplementation improved insulin metabolism markers and gene expression in IVF candidates.
13	Di Bari et al. (2021) (54), Italy	Review	–	Not applicable	Bone metabolism, fracture risk in PCOS	–	Low Vitamin D associated with bone risks in PCOS, indirectly impacting reproductive health.
14	Domingues et al. (2019) (55), Brazil	Case–Control	20 PCOS, 20 oocyte donors	Endogenous Vitamin D in follicular fluid	Proteomic profile of FF	Mass spectrometry	Distinct protein expression related to Vitamin D observed in PCOS patients.
15	Farhangnia et al. (2024) (56), Iran	Narrative Review	–	Endometriosis and Vitamin D	Role in endometrial environment	–	Vitamin D deficiency associated with inflammation and poor endometrial receptivity.
16	Fernando et al. (2020) (57), Australia	Review	–	VDBP and pregnancy outcomes	Mechanisms in Vitamin D transport	–	VDBP levels affect Vitamin D bioavailability in pregnancy and IVF.
17	Grzeczka et al. (2022) (15), Poland	Review	–	Focus on ovarian follicle	Role in oocyte maturation	–	Vitamin D important for follicular development; deficiency impairs oocyte quality.
18	Hager et al. (2019) (58), Austria	Retrospective Cohort	62 women	Incidental endometriosis and Vitamin D	Endometriosis prevalence	Serum 25(OH)D	Women with PCOS and endometriosis had lower Vitamin D levels.
19	Hu et al. (2020) (59), China	RCT protocol	–	Pre-IVF Vitamin D supplementation	IVF pregnancy rates	To be measured	Trial to assess Vitamin D before IVF in PCOS population.
20	Hu et al. (2023) (3), China	Prospective Cohort	112 women with endometriosis	Serum 25(OH)D3	IVF outcomes	ELISA	Higher Vitamin D levels linked with better IVF outcomes via granulosa cell proliferation.
21	Iervolino et al. (2021) (28), Italy	Review	–	Natural molecules incl. Vitamin D	Fertility in PCOS	–	Vitamin D proposed as adjunctive treatment in PCOS.
22	Irani et al. (2017) (29), USA	RCT	60 women with PCOS	50,000 IU Vitamin D biweekly	Serum VEGF, clinical PCOS symptoms	ELISA	Vitamin D reduced VEGF and improved symptoms in PCOS.
23	Jeon (2023) (30), Korea	Narrative Review	–	Vitamin D and AMH	Ovarian reserve, depression	–	Low Vitamin D linked to low AMH and higher depressive symptoms.
24	Kalyanaraman and Pal (2021) (17), USA	Review	–	Pathophysiology of PCOS	Vitamin D pathways	–	Emphasizes role of Vitamin D in insulin resistance and inflammation in PCOS.
25	Ko et al. (2022) (2), Hong Kong	Retrospective Cohort	874 women	Serum 25(OH)D before IVF	Cumulative live birth rate	Immunoassay	Higher Vitamin D predicted higher cumulative live birth rates.
26	Kolcsar et al. (2024) (31), Hungary	Cross-sectional	220 women	Serum 25(OH)D	AMH levels in non-PCOS IVF	Immunoassay	Vitamin D positively correlated with AMH.
27	Meng et al. (2023) (75), Hungary	Retrospective	192 infertile women	Serum 25(OH)D	Hormonal status	LC–MS/MS	Lower Vitamin D linked with hormonal dysregulation.
28	Kotlyar and Seifer (2023) (33), USA	Review	-	IVF in PCOS	Overview of therapies	–	Vitamin D among adjuvant strategies.
29	Lerchbaum et al. (2021) (11), Austria	RCT	180 PCOS women	20,000 IU/week Vitamin D	Androgens, AMH	ECLIA	No significant effect found on AMH, but trend toward improved androgens.
30	Li et al. (2024) (9), China	Review	–	Vitamin D3 and follicle development	Ovarian physiology	–	Vitamin D supports folliculogenesis.
31	Luddi et al. (2016) (34), Italy	Prospective	40 IVF patients	Endogenous Vitamin D in FF	Oxidative stress and IVF outcome	LC–MS/MS	Antioxidants and Vitamin D reduced oxidative stress in FF of aged women.
32	Maaherra Armstrong et al. (2023) (35), Sweden	Cross-sectional	420 IVF patients	Serum 25(OH)D	Vitamin D insufficiency prevalence	Immunoassay	High prevalence of insufficiency; correlated with BMI and lifestyle factors.
33	Mancini et al. (2021) (36), Italy	Review	–	Oxidative stress and inflammation	PCOS mechanisms	–	Vitamin D modulates inflammatory and oxidative pathways in PCOS.
34	Meng et al. (2023) (37), China	Meta-analysis	12 studies	Vitamin D supplementation	Clinical pregnancy and live birth rates	–	Supplementation improved pregnancy and live birth rates.
35	Mesinovic et al. (2020) (38), Australia	Cross-sectional	51 PCOS patients	25(OH)D and D metabolites	Androgen levels	LC–MS/MS	Vitamin D metabolites inversely correlated with androgens.
36	Morgante et al. (2022) (18), Italy	Review	–	Vitamin D and PCOS	Physiology and treatment	–	Supports Vitamin D role in PCOS pathophysiology and therapy.
37	Nandakumar et al. (2024) (5), UK	Cross-sectional	108 women	Vitamin D status	Cardiovascular biomarkers	Immunoassay	Low Vitamin D associated with adverse CV biomarkers in PCOS.
38	Nandi et al. (2016) (39), USA	Review	–	Vitamin D and fertility	Mechanisms of action	–	Supports role in hormonal and metabolic regulation.
39	Omran et al. (2020) (14), Egypt	Prospective Cohort	100 PCOS undergoing ICSI	Serum 25(OH)D	ICSI outcomes	ELISA	Higher Vitamin D levels associated with better fertilization and pregnancy rates.
40	Ota et al. (2023) (40), Japan	Cross-sectional	207 infertile women	Serum 25(OH)D	Reproductive and immune markers	Immunoassay	Seasonal variations of Vitamin D influence immune markers.
41	Paffoni et al. (2019) (41), Italy	RCT Protocol	Planned *N* = 120	25(OH)D before/during ART	ART outcomes (pregnancy/live birth)	Serum assay (planned)	Study protocol to assess Vitamin D supplementation impact on ART outcomes.
42	Pagliardini et al. (2015) (12), Italy	Cross-sectional	335 infertile women	Serum 25(OH)D	Prevalence of hypovitaminosis D	Immunoassay	High prevalence of Vitamin D deficiency; associated with infertility.
43	Piao et al. (2024) (8), China	Retrospective & Prospective	357 PCOS patients	Vitamin D levels & supplementation	Pregnancy outcomes	Serum assay	Supplementation improved pregnancy rates in Vitamin D-deficient women.
44	Rogenhofer et al. (2022) (42), Germany	Retrospective Cohort	482 IVF/ICSI patients	Seasonal serum 25(OH)D	AMH, ovarian response	Immunoassay	Seasonal variation in Vitamin D levels linked to AMH and IVF response.
45	Saeed et al. (2022) (1), India	Systematic Review	12 studies	Vitamin D in treatment of OLP	Therapeutic effectiveness	-	Findings suggest potential role for Vitamin D, though not specific to IVF/PCOS.
46	Schroder-Heurich and Von Versen-Hoynck (76), Germany	Narrative Review	–	Vitamin D deficiency in fertility	Mechanistic insight	–	Highlights link between Vitamin D and reproductive hormones.
47	Shahrokhi et al. (2016) (44), Iran	Narrative Review	–	Vitamin D in reproduction	Follicular development and endometrial receptivity	–	Emphasizes role of Vitamin D in endometrial receptivity and fertility.
48	Shen et al. (2017) (45), China	Proteomic Analysis	40 IVF patients	Vitamin D in follicular fluid	IVF success-related proteins	Mass spectrometry	Certain proteins related to Vitamin D status linked to IVF success.
49	Showell et al. (2018) (46), International	Cochrane Review	10 RCTs	Inositol in PCOS	IVF outcomes, ovulation, live birth	–	Inositol may improve IVF outcomes; indirect support for Vitamin D adjuncts.
50	Singh et al. (2023) (47), India	Review	–	PCOS pathogenesis	Therapeutic strategies including Vitamin D	–	Supports Vitamin D as part of PCOS management due to anti-inflammatory effects.
51	Skowrońska et al. (2016) (48), Poland	Systematic Review	–	Vitamin D and reproductive dysfunction	Fertility and IVF outcomes	–	Low Vitamin D associated with reproductive dysfunction, especially in PCOS.
52	Sparic et al. (2024) (6), Serbia	Review	–	Vitamin D in PCOS	Clinical impact of Vitamin D	–	Clinical data support the link between Vitamin D status and PCOS symptoms.
53	Süli et al. (2023) (49), Hungary	Experimental Animal Study	Rat model of PCOS	Vitamin D supplementation in PCOS rats	Vascular reactivity	Serum 25(OH)D	Vitamin D had a protective vascular effect in a PCOS model, especially in females.
54	Vanni et al. (2014) (50), Italy	Review	–	Vitamin D in ART	ART outcomes, follicular development	–	Highlights positive associations of Vitamin D with ART success.
55	Várbíró et al. (2022) (16), Hungary	Review	–	Fertility, pregnancy, PCOS	Role of Vitamin D	–	Summarizes clinical and preclinical findings supporting Vitamin D’s role in fertility.
56	Wesselink et al. (2015) (51), USA	Narrative Review	–	Supplements in IVF	IVF outcomes	–	Discusses multiple supplements including Vitamin D as beneficial for IVF.
57	Wu et al. (2019) (13), USA	Observational	103 IVF patients	Serum 25(OH)D	Ovarian response, inflammatory markers	ELISA	Vitamin D deficiency associated with poor ovarian response and pro-inflammatory profile.
58	Zhang et al. (2022) (52), China	Prospective Cohort	1,062 couples	Serum Vitamin D before conception	Time to pregnancy, pregnancy outcomes	LC–MS/MS	Higher Vitamin D levels associated with shorter time to pregnancy and better outcomes.
59	Zhou et al. (2022) (4), China	Meta-analysis	5 RCTs	Vitamin D supplementation	IVF outcomes	–	Supplementation improves clinical pregnancy rate and may reduce miscarriage risk.

### Summary of findings key studies on vitamin D and IVF outcomes in PCOS

1. Impact of vitamin D on IVF outcomes:
  - Ko et al. (2) found that higher serum vitamin D levels before ovarian stimulation were associated with increased cumulative live birth rates in women undergoing IVF.
  - Hu et al. (3) demonstrated that 25(OH)D3 improved granulosa cell proliferation, which is crucial for successful IVF outcomes in patients with endometriosis, suggesting potential implications for PCOS patients.
  - Zhou et al. (4) conducted a meta-analysis showing a positive effect of vitamin D supplementation on IVF outcomes, reinforcing the potential benefits of optimal vitamin D levels.
2. Vitamin D and PCOS:
  - Nandakumar et al. (5) explored cardiovascular risk biomarkers in non-obese women with and without PCOS, highlighting an association with vitamin D levels.
  - Sparic et al. (6) provided a clinical appraisal of the role of vitamin D in PCOS, suggesting that vitamin D deficiency might exacerbate PCOS symptoms and impact fertility.
3. Biological Mechanisms:
  - Bakhshalizadeh et al. (7) studied the modulation of steroidogenesis by vitamin D3 in granulosa cells, indicating a mechanistic pathway through which vitamin D might affect reproductive outcomes in PCOS.
  - Moridi et al. (72) reviewed the association between vitamin D and anti-Müllerian hormone, a marker of ovarian reserve, suggesting potential benefits of vitamin D in improving ovarian function.

### Distribution of papers based on years (see Table 4)

**Table 4:** Distribution of reviewed papers by year.

Year	Number of papers
2013	1
2014	1
2015	3
2016	4
2017	3
2018	2
2019	4
2020	5
2021	6
2022	8
2023	7
2024	8

The systematic literature review included a total of 59 studies, spanning from 2013 to 2024. The distribution of these studies over the years is illustrated in Table 1. This distribution highlights the growing interest and research focus on the role of vitamin D in IVF outcomes for PCOS patients over the past decade (see Table 3).

An analysis of the publication trends reveals a significant rise in research output starting in 2019, with the highest number of studies published in 2022 and 2024. This upward trend reflects a growing acknowledgment of the critical role of vitamin D in reproductive health, particularly concerning IVF and PCOS. In both 2022 and 2024, eight studies were published, marking a peak in research activity. This increase may be linked to advancements in understanding the biochemical and physiological functions of vitamin D in fertility and its potential to enhance IVF outcomes in women with PCOS. The findings underscore the importance of sustained research efforts to uncover the underlying mechanisms and therapeutic potential of vitamin D supplementation in improving reproductive outcomes for PCOS patients undergoing IVF.

### Distribution of papers based on developing countries (see Table 5)

**Table 5:** Distribution of studies by country.

Country	Number of studies	Percentage of total studies
China	3	10%
India	2	6.7%
Egypt	1	3.3%
Brazil	1	3.3%
Iran	2	6.7%
Other developing	0	0%
Developed countries	21	70%

In this systematic literature review, we evaluated studies exploring the association between baseline vitamin D levels and *in vitro* fertilization (IVF) outcomes in patients with polycystic ovary syndrome (PCOS). A key component of our analysis involved examining the geographic distribution of the research, with particular attention to contributions from developing countries. This focus is essential for understanding how regional variations in healthcare systems and vitamin D prevalence may impact the findings.

The majority of studies (70%) were conducted in developed countries, reflecting a concentration of research resources and infrastructure. However, there is a notable representation from developing countries such as China, India, and Iran, which collectively account for 26.7% of the studies. This indicates a growing interest and capability in these regions to conduct research on fertility and vitamin D, potentially driven by the high prevalence of PCOS and vitamin D deficiency in these populations.

The pattern of study distribution indicates a disparity in research activity between developed and developing nations. Although developed countries dominate the research landscape, studies from developing regions are critical for providing insights tailored to specific contexts. These findings can guide the development of targeted strategies to enhance IVF outcomes for PCOS patients in various healthcare settings. To achieve a more holistic understanding of the global effects of vitamin D on fertility, future research should focus on improving representation from underrepresented regions.

### Relationship between vitamin D, IVF, and PCOS

The systematic review included 59 studies, with a focus on 10 key studies that directly examined the relationship between vitamin D levels and IVF outcomes in PCOS patients. The findings are summarized in Table 1 and Figure 1 (see Table 6).

**Table 6:** Summary of key studies on vitamin D, IVF, and PCOS.

Study	Population	Vitamin D measurement	IVF outcome	Key findings
Ko et al. (2)	Women undergoing IVF	Serum 25(OH)D	Cumulative live birth rate	Higher vitamin D levels correlated with increased live birth rates.
Hu et al. (3)	Endometriosis patients	25(OH)D3	Granulosa cell proliferation	Improved pregnancy outcomes with higher vitamin D levels.
Piao et al. (8)	PCOS patients	Serum vitamin D	Pregnancy rates	Positive association between vitamin D levels and pregnancy success.
Zhou et al. (4)	General IVF patients	Vitamin D supplementation	IVF success rate	Vitamin D supplementation improved IVF outcomes.
Omran et al. (14)	PCOS patients	Serum vitamin D	ICSI outcome	Higher vitamin D levels were associated with better ICSI outcomes.

Analysis:

1. Vitamin D levels and IVF outcomes
  - Several studies, including Ko et al. (2) and Piao et al. (8), demonstrated a significant positive correlation between serum vitamin D levels and successful IVF outcomes, such as increased live birth and pregnancy rates in PCOS patients.
  - Zhou et al. (4) highlighted the benefits of vitamin D supplementation, suggesting enhanced IVF success rates across various patient groups.
2. Granulosa cell function and follicular development
  - Hu et al. (3) highlighted that 25(OH)D3 enhances granulosa cell proliferation, which is crucial for follicular development, thereby potentially improving IVF outcomes.
  - Li et al. (9) supported these findings by emphasizing the role of vitamin D3 in follicle development, suggesting that adequate vitamin D levels may promote optimal follicular growth and maturation.
3. Hormonal and metabolic influences
  - Dastorani et al. (10) reported that vitamin D supplementation positively affected metabolic profiles and gene expression related to insulin and lipid metabolism in PCOS patients, which could indirectly improve reproductive outcomes.
  - Lerchbaum et al. (11) observed that vitamin D supplementation improved surrogate markers of fertility, such as anti-Müllerian hormone (AMH) levels, in women with PCOS.
4. Vitamin D deficiency prevalence
  - Pagliardini et al. (12) documented a high prevalence of vitamin D deficiency among infertile women seeking assisted reproduction, underscoring the importance of addressing this deficiency to optimize fertility treatments.
5. Immunological and inflammatory factors
  - Wu et al. (13) found that low serum vitamin D levels were associated with pro-inflammatory immune responses, which could negatively impact IVF outcomes. Adequate vitamin D levels might mitigate these inflammatory responses, enhancing fertility.
6. Impact on PCOS patients
  - The studies specifically focusing on PCOS patients, such as Omran et al. (14), indicated that higher serum vitamin D levels were linked to improved outcomes in assisted reproductive technologies like ICSI.
  - Vitamin D’s role in modulating granulosa cell function and reducing oxidative stress, as shown by Hu et al. (3), further supports its positive impact on reproductive health in PCOS patients.
7. Biological mechanisms:
  - The potential mechanisms through which vitamin D influences IVF outcomes include modulation of ovarian function, enhancement of endometrial receptivity, and reduction of systemic inflammation, as discussed in several studies.
8. Clinical implications
  - These findings underscore the importance of assessing and optimizing vitamin D levels in women undergoing IVF, particularly those with PCOS, to improve reproductive outcomes.
  - Further research is needed to establish standardized guidelines for vitamin D supplementation in this patient population.

### Visual data summary of key study outcomes (see Table 7)

**Table 7:** Summary of key study outcomes.

Study	Outcome	Vitamin D influence
Ko et al. (2)	Cumulative live birth rate	Positive correlation with vitamin D levels
Piao et al. (8)	Pregnancy rates	Increased with vitamin D supplementation
Hu et al. (3)	Granulosa cell proliferation	Enhanced by 25(OH)D3
Dastorani et al. (10)	Metabolic profiles	Improved with vitamin D supplementation
Pagliardini et al. (12)	Vitamin D deficiency prevalence	High among infertile women

The table provides a clear overview of the various ways vitamin D impacts IVF outcomes in PCOS patients, highlighting the significance of maintaining sufficient vitamin D levels to improve reproductive success. Consistent evidence from multiple studies indicates that vitamin D is vital for several biological processes associated with fertility, such as hormone regulation, cell growth, and metabolic function. Managing vitamin D deficiency may serve as a crucial approach to enhancing IVF outcomes in PCOS patients.

## Discussion

### Vitamin D levels and IVF outcomes

The studies have highlighted the significant role of vitamin D in improving reproductive outcomes in women undergoing fertility treatments, particularly in IVF and those with PCOS. Ko et al. (2) found that vitamin D deficiency was associated with lower Cumulative Live Birth Rates (CLBR) and poorer IVF outcomes, even after adjusting for factors like age and BMI. Additionally, women with vitamin D deficiency required higher doses of gonadotropins and had fewer oocytes retrieved and fertilized normally. The fat-soluble nature of vitamin D may explain its reduced availability in women with higher BMI, further complicating reproductive success. Further research by Piao et al. (8) on women with PCOS revealed that vitamin D deficiency was linked to lower pregnancy rates, while vitamin D supplementation improved pregnancy outcomes, including the regulation of hormonal imbalances associated with PCOS. Lastly, Zhou et al. (4) in their meta-analysis confirmed that vitamin D supplementation could improve chemical pregnancy rates in women with vitamin D deficiency undergoing IVF. Taken together, these studies underscore the potential of vitamin D management, whether through supplementation or monitoring, as an effective strategy for improving fertility and pregnancy outcomes, particularly in women facing vitamin D deficiency or reproductive health challenges such as PCOS.

Vitamin D deficiency during pregnancy has been associated with an increased risk of preeclampsia, gestational diabetes, low birth weight, and impaired fetal bone development. Research by Mariam, et al. (2024) found that vitamin D plays a crucial role in immune regulation, calcium-phosphorus metabolism, and fetal development. Vitamin D, known as a pleiotropic hormone, plays a vital role in calcium and phosphorus metabolism, as well as in regulating immune responses and inflammation. A consistent association has been found between low 25(OH)D levels and an increased risk of preeclampsia, a serious pregnancy complication characterized by hypertension and proteinuria after 20 weeks of gestation. Recent meta-analyses have even shown that vitamin D supplementation can significantly reduce the risk of developing preeclampsia. Furthermore, glucose metabolism disorders during pregnancy may also be exacerbated by low vitamin D levels due to its role in insulin sensitivity. Regarding low birth weight, although some studies report inconsistent findings, meta-analyses still support a negative association between maternal vitamin D deficiency and an increased risk of delivering infants with low birth weight or being small for gestational age (SGA). In addition, since vitamin D is essential for bone formation, its deficiency may impair fetal skeletal development and lead to bone growth disorders.

### Granulosa cell function and follicular development

The original research Hu, et al. (3) showed that 25(OH)D3 enhanced the proliferation capacity of granulosa cells, particularly at a concentration of 10 nM. This suggests that adequate vitamin D levels may support ovarian cell division, which is important for follicular development and oocyte maturation. Moreover 25(OH)D3 increased the proportion of cells in the G2M + S phases of the cell cycle, promoting progression and division. Also observed that 25(OH)D3 altered the expression of several key genes, such as CDKN2D (downregulated) and TGFB2 and THBD (upregulated), which are involved in cell cycle regulation and ovarian function. The clinical analysis showed that endometriosis patients with sufficient levels of 25(OH)D3 (≥20 μg/mL) had better IVF outcomes compared to those with vitamin D deficiency. These patients had higher embryo quality, more embryos available for transfer, and better pregnancy rates. Specifically, adequate levels of vitamin D were found to be a protective factor for live birth outcomes. The study found that 25(OH)D3 levels were lower in endometriosis patients compared to controls, and that vitamin D deficiency was linked to poor IVF outcomes. Adequate 25(OH)D3 levels improved granulosa cell proliferation and promoted cell cycle progression, increasing the G2M + S phase cells. The study also identified altered gene expressions (e.g., CDKN2D downregulated, TGFB2 and THBD upregulated) that may contribute to these effects. Adequate 25(OH)D3 was a protective factor for better IVF outcomes, including embryo quality and live birth rates. The review study Li et al. (9) showed that Vitamin D3 plays an important role in follicular development, which is closely related to female reproductive system diseases and fertility. Vitamin D3 is locally synthesized within follicles and its receptor Vitamin D receptors (VDR) is widely distributed in follicles, indicating its important role in follicular development. The hormone plays a role in the transition from primordial follicle activation to dominant follicle formation, as well as in the later stages of follicular growth and maturation. Vitamin D3 promotes granulosa cell (GC) proliferation and supports follicle growth and also helps regulate molecules involved in oocyte meiosis and the secretion of steroid hormones by granulosa cells (GCs). Vitamin D3 regulates genes involved in the cell cycle to promote GC proliferation. Additionally, it exhibits antioxidant and anti-apoptotic effects, offering protection to follicles. Vitamin D3 supplementation can improve the *in vitro* maturation (IVM) rate of oocytes, particularly in the context of oocyte cryopreservation. It enhances follicle development in ART by supporting the culture of oocytes before and after cryopreservation, improving the success rate of IVF procedures.

### Hormonal and metabolic influences

A randomized controlled trial by Dastorani et al. (10) found that Vitamin D supplementation (50,000 IU every other week for 8 weeks) significantly improved insulin sensitivity, as evidenced by decreased insulin levels and a lower Homeostasis Model Assessment of Insulin Resistance (HOMA-IR) score. The treatment group also experienced reductions in total and LDL cholesterol. Additionally, serum AMH (Anti-Müllerian Hormone), a marker of ovarian reserve, decreased with vitamin D supplementation. However, there were no changes in fasting glucose or triglyceride levels between the treatment and placebo groups. Another randomized controlled trial by Lerchbaum et al. (11) showed that Vitamin D supplementation (20,000 IU per week for 24 weeks) significantly lowered the LH/FSH ratio and increased FSH levels in women with PCOS. A disturbance in the secretion of gonadotropin-releasing hormone leads to a relative increase in LH compared to FSH. This imbalance, along with abnormal ovarian estrogen levels, disrupts the feedback mechanism, resulting in elevated LH secretion. An elevated LH/FSH ratio is often seen in PCOS and contributes to anovulation in many patients. Research suggests that vitamin D may influence FSH sensitivity and plays a role in ovarian follicle development and luteinization. In induced PCOS rats, vitamin D treatment enhanced the number of normal follicles by increasing FSH and estradiol levels while decreasing LH.

### Vitamin D deficiency prevalence

Cross sectional analysis (12) showed 89% of infertile women seeking assisted reproduction had serum 25(OH)D levels ≤30 ng/mL, with 48% having levels ≤20 ng/mL, indicating a widespread deficiency. Deficiency was more common in women with lower BMI and those with endometriosis. Sun exposure and body composition were significant factors influencing vitamin D levels. The findings highlight the importance of addressing vitamin D deficiency in infertile women, as it may be linked to reproductive health and IVF outcomes. The study found that vitamin D deficiency negatively impacts the clinical pregnancy rate in assisted reproductive technology (ART), suggesting that correcting vitamin D deficiency could provide new therapeutic approaches for women undergoing IVF and potentially for all women experiencing infertility.

### Immunological and inflammatory factors

A retrospective cross-sectional study by Wu et al. (13) involving 157 women with IVF failures found that low serum vitamin D levels (<30 ng/mL) were strongly associated with poor ovarian response (POR) and heightened pro-inflammatory immune responses. Women with low vitamin D and POR exhibited significantly elevated levels of peripheral blood natural killer (NK) cells, increased NK cell cytotoxicity, and a higher T helper 1 (Th1) to T helper 2 (Th2) cytokine ratio (as measured by TNF-*α*/IL-10 and IFN-*γ*/IL-10). These findings suggest a dominance of pro-inflammatory immune pathways. Additionally, these women had elevated levels of homocysteine and plasminogen activator inhibitor-1 (PAI-1), both metabolic markers linked to inflammation and poor vascular health. The combination of these immune and metabolic dysregulations was correlated with impaired ovarian folliculogenesis and reduced oocyte quality during IVF cycles. While vitamin D acts as an immunomodulator and anti-inflammatory agent, supplementation strategies were proposed to potentially mitigate these abnormalities and improve IVF outcomes.

### Impact on PCOS patient

Vitamin D levels play a significant role in assisted reproductive technology (ART) outcomes, particularly in conditions like PCOS and endometriosis. A study by Omran et al. (14) highlighted that higher serum vitamin D levels in PCOS patients undergoing ICSI were associated with improved ovarian response, as evidenced by a greater number of retrieved and fertilized oocytes. Specifically, each 2 ng/mL increase in vitamin D corresponded to one additional retrieved oocyte, while a 3 ng/mL increase led to one more fertilized oocyte. This association persisted even after adjusting for confounding factors such as age and BMI. Vitamin D’s role in regulating key reproductive hormones like AMH and FSH, as well as its anti-inflammatory effects, is thought to support these improvements. However, no significant correlation was found between vitamin D levels and pregnancy rates in this group. Similarly, Rui Hu et al. (3) demonstrated that adequate levels of 25(OH)D3 in follicular fluid (≥20 ng/mL) significantly improved embryo quality, the number of transferable embryos, and clinical pregnancy rates in patients with endometriosis.

Mechanistically, 25(OH)D3 enhanced granulosa cell proliferation by promoting cell cycle progression, increasing cells in the active G2M + S phases while upregulating THBD and downregulating CDKN2D expression. This cellular effect directly supported follicular growth and oocyte quality, ultimately improving live birth outcomes. Together, these findings suggest that vitamin D sufficiency positively impacts ART outcomes by enhancing ovarian function, granulosa cell proliferation, and oocyte quality, particularly in challenging conditions like PCOS and endometriosis.

### Biological mechanism

Several studies have explained the potential mechanisms through which vitamin D influences IVF outcomes in PCOS patients. A study by Grzeczka et al. (15) highlighted that vitamin D plays a crucial role in ovarian function through its influence on folliculogenesis and oocyte maturation. Granulosa cells, which are essential for follicular development, express vitamin D receptors (VDRs), and their activation by vitamin D enhances steroidogenesis. Specifically, vitamin D regulates genes involved in the production of sex steroids, such as aromatase (CYP19A1), leading to increased production of estradiol and progesterone, hormones vital for follicle maturation and oocyte quality. Another study by Moridi et al. (72) systematically reviewed the association between vitamin D and ovarian reserve markers, finding that vitamin D supplementation significantly increased anti-Müllerian hormone (AMH) levels in ovulatory women without PCOS. AMH is a critical marker of ovarian reserve, and its elevation suggests improved ovarian responsiveness during IVF. In contrast, vitamin D deficiency impairs these pathways, leading to poor follicular development, lower oocyte quality, and diminished IVF outcomes. These findings underscore the importance of adequate vitamin D levels for optimizing ovarian function and ART success.

Pich et al. (73) conducted an experimental study on PCOS-induced rats and found that vitamin D supplementation improved endometrial receptivity by modulating key uterine adipokines and adhesion molecules. In vitamin D-treated rats, levels of chemerin and adiponectin, adipokines linked to inflammation and implantation, were restored to normal levels, suggesting an improvement in the uterine environment. Additionally, vitamin D influenced the expression of HOXA10, a gene critical for endometrial cell differentiation and implantation, and increased integrin levels, which are essential for embryo adhesion to the endometrium. A review by Várbíró et al. (16) emphasized that vitamin D promotes cell cycle regulation in endometrial cells, reducing apoptosis and enhancing endometrial receptivity. These findings demonstrate that vitamin D’s effects extend beyond ovarian function to create a favorable uterine environment, which is critical for successful implantation and pregnancy during ART.

A narrative review by Kalyanaraman and Pal (17) also explained the anti-inflammatory role of vitamin D in ART, particularly for conditions like PCOS and endometriosis. Vitamin D was shown to suppress pro-inflammatory cytokines such as tumor necrosis factor-alpha (TNF-*α*) and interleukin-6 (IL-6) while promoting regulatory T-cell (Treg) activity. This shift from a pro-inflammatory to an anti-inflammatory state reduces chronic inflammation in reproductive tissues, improving ovarian and endometrial function. Another study by Morgante et al. (18) highlighted that vitamin D supplementation lowered markers of systemic inflammation, which are often elevated in PCOS patients. By reducing Th1 and Th17 dominance, vitamin D creates a more balanced immune environment that supports implantation and embryo development. These mechanisms are particularly beneficial in ART, as inflammation is a significant barrier to successful outcomes, especially in patients with underlying inflammatory conditions.

## Strengths and limitations of the evidence

The strength of the evidence lies in the consistency of findings across various study designs (RCTs, cross-sectional studies, and retrospective analyses) showing a beneficial role of vitamin D in reproductive outcomes. However, limitations include significant heterogeneity in study populations, variations in vitamin D measurement and dosing, and inconsistent definitions of IVF success. Not all findings were statistically significant, and some trials failed to show a clear benefit, highlighting the need for more standardized protocols and better methodological rigor.

### Clinical implications

These findings support the importance of screening for vitamin D deficiency and providing personalized supplementation to improve IVF outcomes, especially in women with PCOS. Assessing vitamin D levels should be part of routine fertility evaluations and pre-IVF preparation.

### Future research directions

Future studies should determine the optimal dose and timing of vitamin D supplementation in IVF cycles. Stratified research based on baseline vitamin D levels and large multicenter trials are needed to confirm causality and guide clinical practice. Greater inclusion of diverse populations will enhance the generalizability of results.

## Conclusion

The relationship between initial vitamin D levels and *in vitro* fertilization (IVF) outcomes in women with polycystic ovary syndrome (PCOS) suggests that vitamin D plays a crucial role in enhancing IVF success, although the findings remain somewhat inconsistent. Research generally points to a positive correlation between higher baseline vitamin D levels and improved reproductive results, including increased live birth rates, pregnancy rates, and better ovarian responses during IVF treatments.

However, despite this supporting evidence, there is still variation across different study designs, populations, and supplementation protocols. The specific biological mechanisms by which vitamin D affects fertility are still under investigation, with possible pathways involving the regulation of ovarian function, enhancement of follicular growth, and improved endometrial receptivity. Given the common occurrence of vitamin D deficiency among women with PCOS, which is associated with poorer IVF outcomes, regulating vitamin D levels could be a key factor in boosting fertility outcomes for this group.

Considering the mixed results and the necessity for further investigation to establish standardized supplementation protocols, future research should focus on determining the optimal vitamin D levels for successful IVF and creating definitive guidelines for supplementation, particularly for patients with PCOS.

## Data Availability

The original contributions presented in the study are included in the article/supplementary material, further inquiries can be directed to the corresponding author.
